# Supplementary material for: DMRforPairs: identifying Differentially Methylated Regions between unique samples using array based methylation profiles
Source: BMC Bioinformatics. 2014 May 15;15:141. doi: 10.1186/1471-2105-15-141 (PMC4046028; doi:10.1186/1471-2105-15-141)
Supplement: Additional file 2 — DMRforPairs output for the comparison of A431-MCF7 and NA17018-NA17105. Please start from the HTML files in each folder. Available via the BMC Bioinformatics website. [file 1471-2105-15-141-S2.zip › 1394847754114233_MOESM2_ESM/A431_MCF7/figures/7174.pdf]

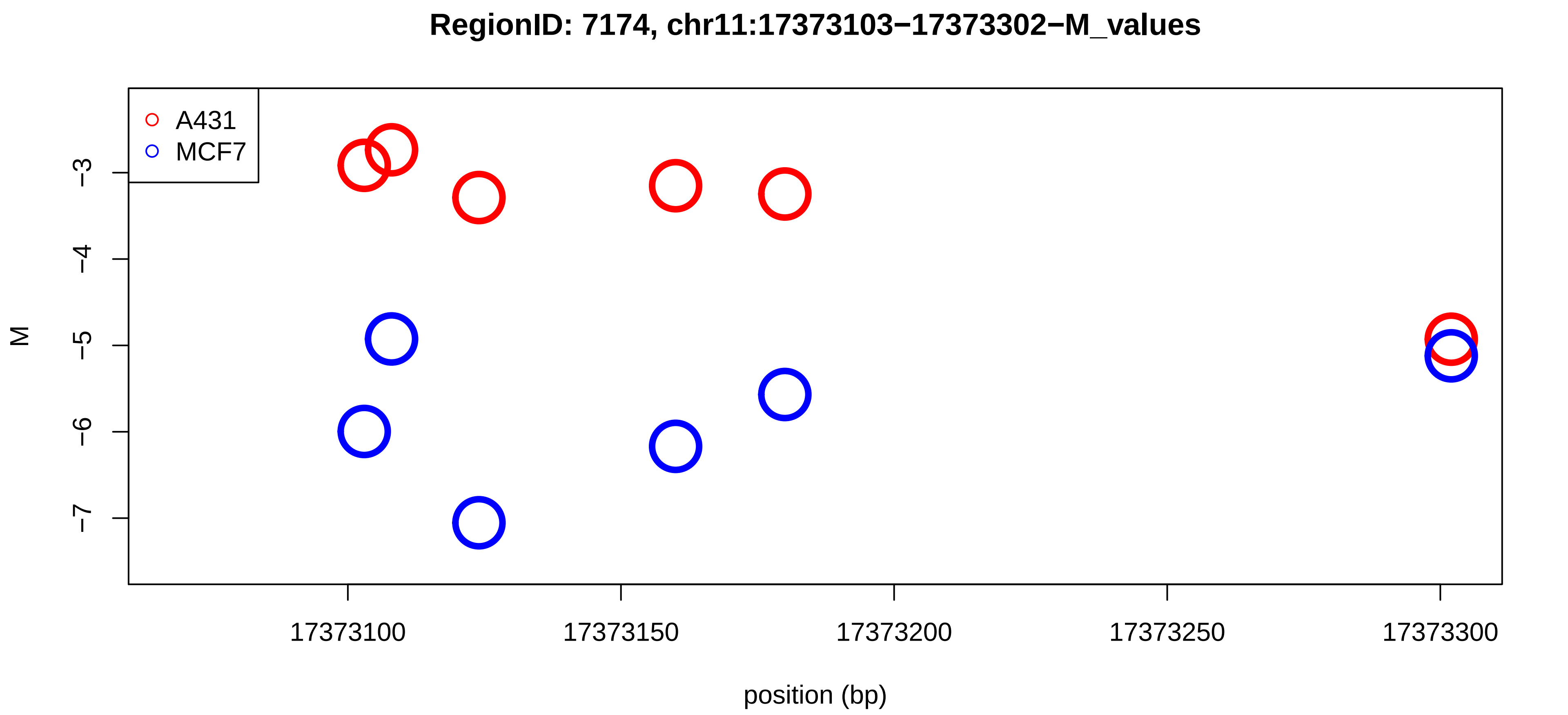
RegionID: 7174, chr11:17373103−17373302−M_values
A431
MCF7
M
−3
−4
−5
−6
−7
17373100
17373150
17373200
17373250
17373300
position (bp)

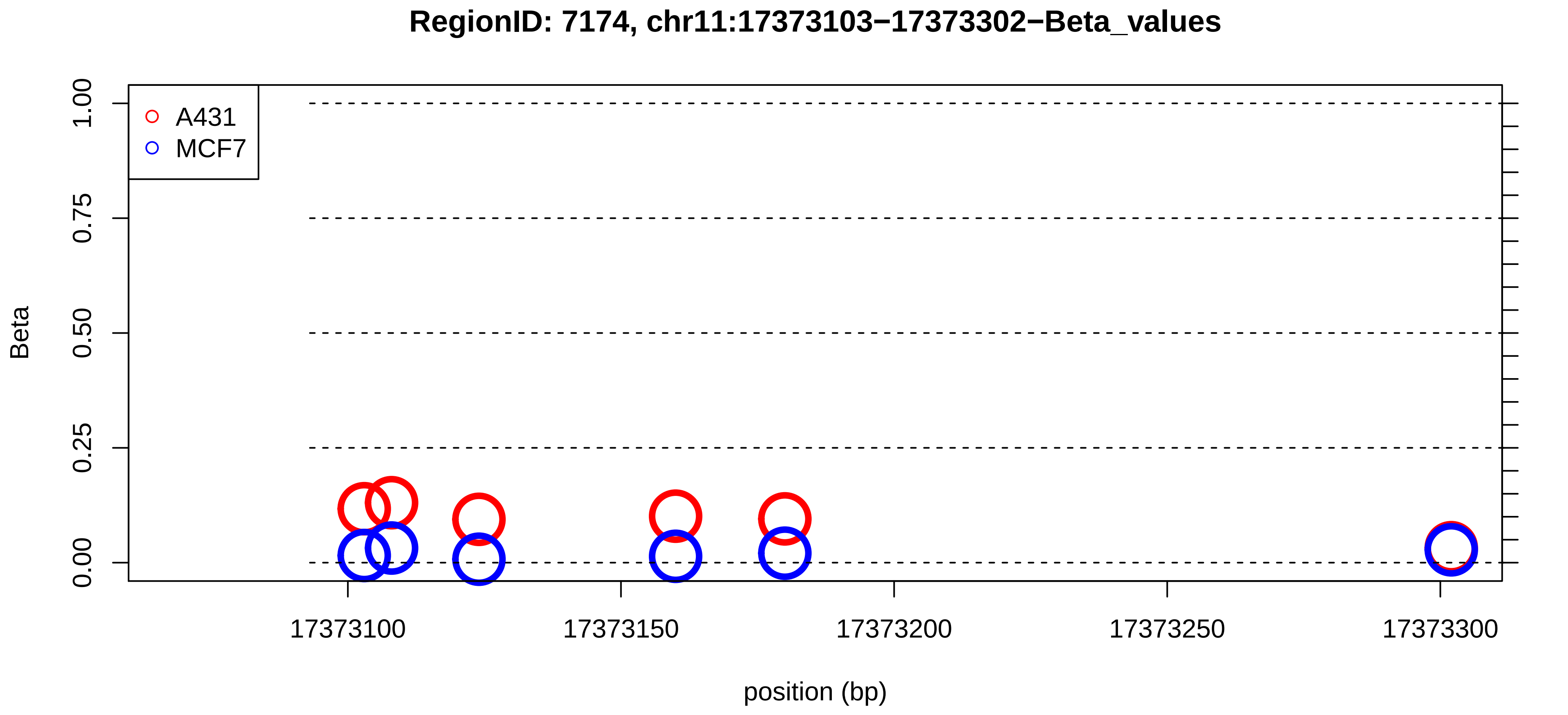
RegionID: 7174, chr11:17373103−17373302−Beta_values
A431
MCF7
Beta
1.00
0.75
0.50
0.25
0.00
17373100
17373150
17373200
17373250
17373300
position (bp)

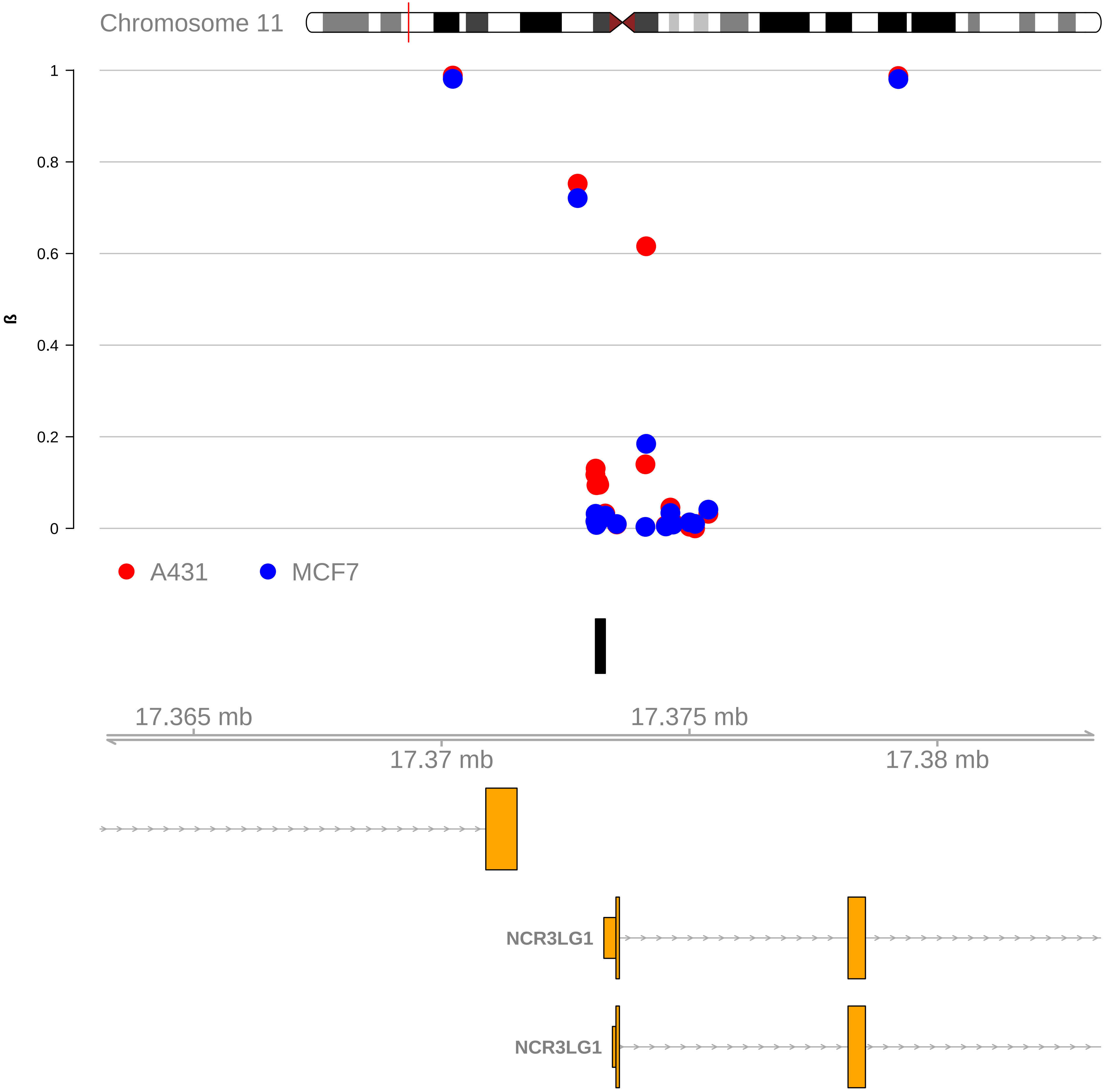
Chromosome 11
1
0.8
0.6
0.4
0.2
0
ß
A431
MCF7
17.365 mb
17.375 mb
17.37 mb
17.38 mb
NCR3LG1
NCR3LG1
